# Embedded Oriented Object Detection on MaixCAM Pro for Waste-Sorting Perception

**DOI:** 10.3390/s26154786

**Published:** 2026-07-28

**Authors:** Kaihan Xie, Ruobing Qin, Jiang Xu, Xiaofei Wang, Yulong Xing

**Affiliations:** 1 Key Laboratory for Advanced Mechatronic System Design and Intelligent Control, School of Mechanical Engineering, Tianjin University of Technology, Tianjin 300384, China; xkh13322@stud.tjut.edu.cn (K.X.); qrb318tt@163.com (R.Q.); xujiang100e01@163.com (J.X.); 2 National Demonstration Center for Experimental Mechanical and Electrical Engineering Education, Tianjin University of Technology, Tianjin 300384, China

**Keywords:** embedded visual sensing, waste sorting, oriented object detection, edge intelligence, post-training quantization, MaixCAM Pro, YOLO11-OBB

## Abstract

For sorting mechanisms that operate on a planar platform, the perception module may need to estimate object category, image-plane position, object extent and principal-axis orientation within the computing budget of a low-power device. This paper reports an oriented object detection workflow for a MaixCAM Pro (Shenzhen Sipeed Technology Co., Ltd., Shenzhen, China) waste-sorting prototype. The sensing output is represented by oriented bounding boxes (OBBs), which provide two-dimensional position, rectangular extent and principal-axis orientation for downstream sorting control. To adapt the detector to a small self-built dataset and INT8 deployment, class- and aspect-ratio-aware re-sampling (CAR-RS) repeats selected training images, while class- and aspect-ratio-aware calibration (CAR-Calib) selects a stratified calibration subset for post-training quantization. The YOLO11-OBB model is exported as a static ONNX graph, adapted by exposing the OBB head output tensors, compiled with TPU-MLIR and executed through native MaixPy OBB inference. On the original PC validation split, the baseline and CAR-RS models both obtain 0.995 mAP50, with mAP50–95 values of 0.935 and 0.932, respectively. CAR-RS reduces the angle mean absolute error from 3.403° to 3.201° for objects with aspect ratio greater than or equal to 1.3. On an additional 80-image cross-background test set that was not used for training or calibration, CAR-RS improves precision from 0.839 to 0.907, recall from 0.882 to 0.893 and mAP50 from 0.916 to 0.927, while mAP50–95 changes from 0.797 to 0.789. On a 100-image MaixCAM Pro validation set, the CAR-RS + CAR-Calib INT8 model achieves 0.961 precision, 0.994 recall, 0.977 F1-score and 0.980 mAP50. The deployed INT8 model occupies 11.85 MB and runs at 8.40 FPS. The results support the technical feasibility of executing an OBB detector on MaixCAM Pro, while also showing that independent-scene validation remains necessary for broader deployment.

## 1. Introduction

Automatic waste sorting machines need a compact visual sensing module that can identify waste categories and provide geometric information for subsequent handling. For sorting mechanisms that operate on a planar platform, the actuator may need more than a class label. It also needs the image-plane position, object extent and principal-axis orientation to drive grasping, tilting or unloading. When objects are randomly placed on a platform, horizontal bounding boxes (HBBs) provide only coarse rectangular envelopes and do not describe the long-axis direction. For elongated or partially adhered objects, a cascade based on HBB detection, segmentation and geometric fitting may be sensitive to low contrast, transparent material and contour extraction errors. The present study uses OBBs as a two-dimensional sensing output rather than as a complete six-degree-of-freedom pose estimate.

Sensor-based sorting has become an important route for improving resource recovery from solid waste. Recent reviews show that non-contact sensors, computer vision and learning-based classifiers are increasingly used in waste sorting lines, but practical systems still need robust perception under limited computing resources [1,2]. For image-based waste recognition, systematic studies have reported rapid growth in deep learning methods and have also highlighted recurring problems: small datasets, class imbalance, large appearance variation and the gap between laboratory accuracy and deployable systems [3,4]. Waste-specific perception studies have investigated segmentation, detection and classification using RGB or RGB-D images [5,6,7]. Recent 2025–2026 journal studies further report task-specific waste detectors for household waste, recyclable waste, plastic sorting and e-waste classification [8,9,10,11,12,13,14]. Recent Sensors articles further report that YOLO-family detectors can be useful for real-time waste detection when the task is framed as a visual sensing problem rather than as offline image classification [15,16,17,18].

Most visual waste-sorting studies return horizontal boxes, masks or class labels. These outputs are useful for recognition, but they do not always provide the in-plane orientation needed by a grasping or unloading mechanism. Oriented object detection has been studied extensively in remote sensing and industrial inspection because an oriented bounding box can describe object location, scale and rotation in one compact representation [19,20,21,22,23]. In waste sorting, the same representation is useful when bottles, cans, radishes, batteries or paper strips appear at arbitrary angles on a platform. In this work, OBB detection is treated as a sensing interface: the detector outputs class, center position, rotated box corners and a principal angle for the sorting controller.

Deploying OBB-based sensing on a low-power sorting machine remains a system problem. Edge intelligence places perception close to the camera, but the model must fit the embedded processor, memory and software toolchain [24,25,26]. First, self-built waste datasets are often imbalanced, and repeated acquisition of a limited number of objects may lead to overly optimistic global mAP values. Long-tailed visual-recognition studies show that re-balancing the training exposure of under-represented classes is a common way to reduce this bias [27]. Second, embedded deployment requires a model graph and output tensors that are compatible with the target runtime. MaixCAM Pro uses the SG2002 platform and the MaixPy runtime; YOLO11-OBB deployment requires static input size, supported operators and expected OBB head outputs [28,29,30]. Third, post-training quantization changes activation ranges and may affect classification, localization and angle regression. Prior work on model compression and neural network quantization shows that deployable vision models should be evaluated by accuracy, model size and runtime speed together [31,32].

This study implements and evaluates an embedded OBB detector on a MaixCAM Pro-based waste-sorting prototype. The work is organized around three questions: how to train a detector from a small and imbalanced self-built dataset, how to expose the ONNX head tensors required by the target runtime, and how much accuracy and speed remain after INT8 deployment. The resulting technical points are as follows:

1. A YOLO11-OBB-based embedded sensing framework is implemented for waste sorting. The output includes object class, center position, OBB size, box corners, confidence and long-axis orientation.
2. A class- and aspect-ratio-aware re-sampling strategy (CAR-RS) is used to increase the training exposure of rare classes and elongated objects without changing the network architecture or adding inference cost.
3. A class- and aspect-ratio-aware calibration strategy (CAR-Calib) is used to select representative images for INT8 post-training quantization.
4. The MaixCAM Pro deployment path adapts YOLO11-OBB ONNX outputs, compiles the model with TPU-MLIR and evaluates native MaixPy OBB inference in terms of accuracy, angle error, model size and FPS.

## 2. Materials and Methods

### 2.1. Embedded Visual Sensing Platform

The experimental platform is built around a MaixCAM Pro (Shenzhen Sipeed Technology Co., Ltd., Shenzhen, China). The device integrates a camera and display unit and uses an SG2002 processor (SOPHGO, Beijing, China). The official MaixCAM hardware documentation lists an INT8 NPU computing capability of 1 TOPS and BF16 support for the SG2002 platform, together with UART peripheral support [33]. In this system, MaixCAM Pro acts as the camera-based sensing node: it acquires the image, performs OBB inference and outputs class, oriented box corners, center position and object angle. The camera module is mounted vertically above the rotating platform so that the field of view covers the sorting area. The raw camera stream is initialized at 640×640 pixels. To avoid including the surrounding structure of the prototype, the program crops a fixed region of interest (ROI) at pixel coordinate (85,140) with a size of 440×450 pixels. This cropped ROI is then resized to the detector input size for inference.

The lower-level controller is an Arduino Mega 2560 (Arduino S.r.l., Monza, Italy). MaixCAM Pro communicates with the controller through UART on /dev/ttyS0 at 115,200 bps. After the detection result is stable for four consecutive frames, the MaixCAM program sends one command line in the following format:

(1) CMD,<mode>,<total>,<arm_x>,<arm_y>,<angle>,<class>,<score>.

**Figure 1:**
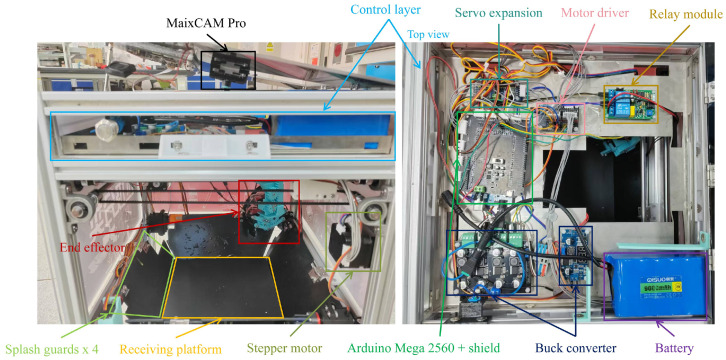
Hardware platform of the MaixCAM Pro-based waste sorting system.

Here, mode is D for direct unloading or G for grasping, total is the number of detected targets, arm_x and arm_y are crop-image coordinates consumed by the Mega2560 program, angle is the OBB principal-axis angle, and class and score are the predicted class ID and confidence. The controller returns READY, BUSY, DONE or ERR. The coordinate mapping used by the prototype is

(2) $$\mathrm{arm}\_x=\mathrm{ROI}\_H-y_{c},\;\mathrm{arm}\_y=x_{c},$$

where $\left(x_{c},y_{c}\right)$ is the detected center in the cropped ROI. This mapping is a prototype-level pixel-to-controller transform. Camera intrinsic calibration and lens-distortion correction were not performed in the current experiments, so the reported quantitative results focus on visual sensing accuracy, angle error and on-device inference speed rather than calibrated metric positioning. The hardware photograph in Figure 1 shows the physical arrangement of the prototype.

**Figure 2:**
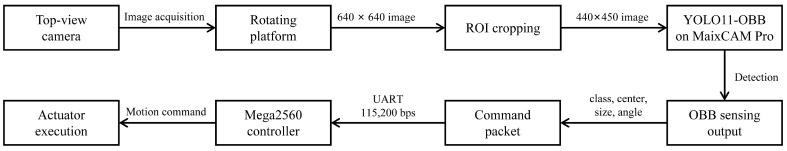
Sensing and command-transfer workflow of the waste sorting system.

The sensing and command-transfer workflow is shown in Figure 2. After image acquisition, the OBB detector returns the object class, oriented box corners and object area. For a single object or same-class objects, the system opens the baffle, moves the gimbal to the target and tilts the platform for sorting. For multiple different waste types, the system computes object areas and class information to determine sorting priority, and the actuator sequentially grasps and releases selected objects. The present work focuses on the embedded visual sensing chain, so the experimental evaluation uses detection accuracy, orientation error, model size and MaixCAM Pro inference speed.

### 2.2. Dataset and Label Format

The experiments use a self-built OBB dataset containing 12 target classes: bottle, cup, battery, metal can, medicine, pebble, brick, potato, carrot, green, red and paper. The object set mixes conventional waste objects with laboratory sorting objects used by the prototype. Bottles, cups, metal cans and paper are mapped to recyclable waste; batteries and medicine packages are mapped to hazardous waste; pebbles and bricks represent other waste; potato, carrot, green and red are food-waste proxy classes used to test visual separation of similar elongated or rounded objects. In the label names, carrot denotes white radish, green denotes green radish, and red denotes carrot or yellow radish. These labels are retained as separate target classes because their appearance and unloading bins are different in the prototype.

**Figure 3:**
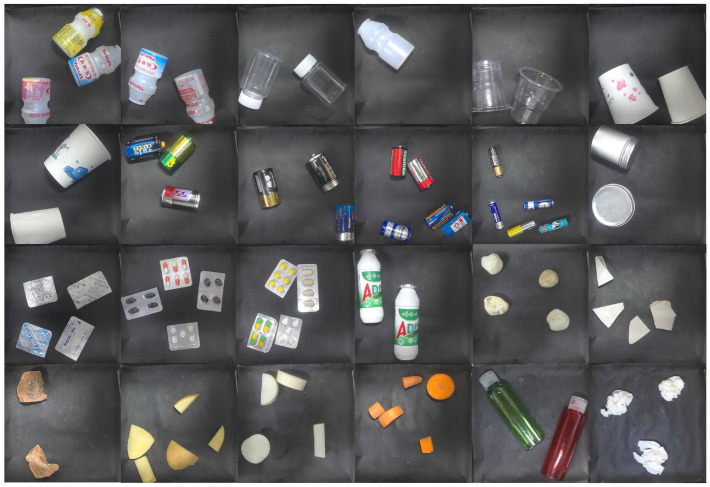
Representative samples from the self-built waste OBB dataset.

The source images were captured by MaixCAM Pro on the sorting platform. During capture, the camera was initialized at 640×640 pixels and the same ROI used by the deployment program was cropped before saving or labeling. The ROI was selected to keep the rotating platform in view and to exclude mechanical parts outside the sorting area. LabelMe 5.9.1 was used to annotate each object with a polygon. The polygon points were then converted to a minimum-area rotated rectangle using OpenCV minAreaRect, and the four corners were written in the YOLO-OBB format [34]. The training set contains 477 images and 1604 labeled objects, while the validation set contains 120 images and 418 labeled objects. Representative samples are shown in Figure 3.

**Figure 4:**
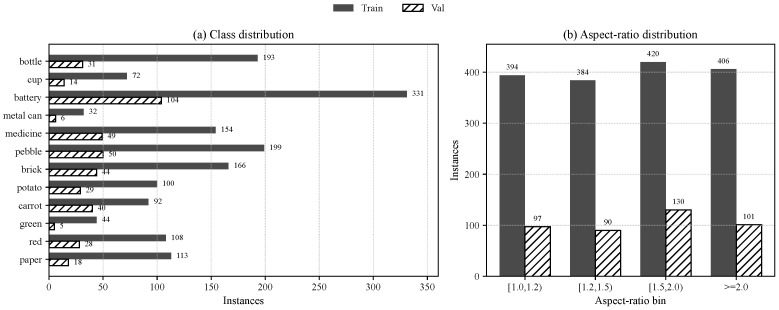
Class instance counts and OBB aspect-ratio distribution of the dataset.

**Figure 5:**
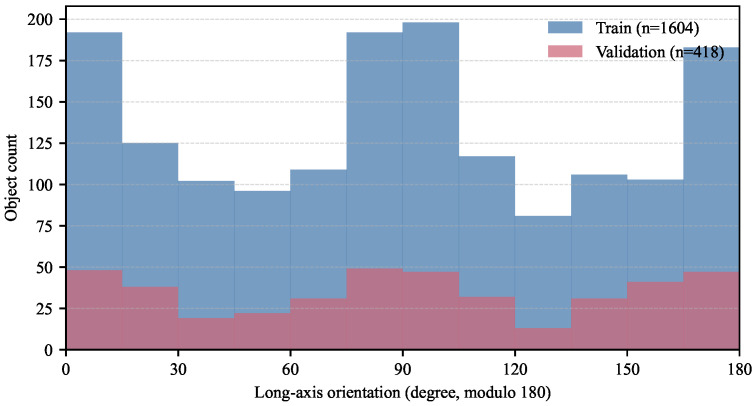
Long-axis orientation distribution of the OBB labels, measured modulo $180^{\circ }$.

Figure 4 shows the class instance counts and OBB aspect-ratio distribution. The dataset is imbalanced, and both the training and validation sets contain many objects with aspect ratio (AR) greater than 1.5. Figure 5 reports the long-axis orientation distribution obtained from the OBB labels. The distribution is not uniform, which is expected for a controlled platform where objects are placed and rotated manually rather than sampled from a large uncontrolled scene. For elongated objects, a small angular deviation may change the estimated principal direction and affect the grasping pose. Therefore, aspect-ratio grouping is used in training, calibration and evaluation.

The original images were collected using fixed equipment and similar scenes. Some classes have repeated object appearances between the training and validation sets, which can make the PC-side validation mAP optimistic. For this reason, the evaluation does not rely only on global mAP. It also reports grouped orientation errors, a 100-image MaixCAM Pro validation test collected and processed through the deployed runtime, and an additional 80-image cross-background test set collected after the first review. The additional test set uses changed backgrounds and moderately different viewing angles; it was not used for training, calibration or model selection. It contains 201 objects from 11 of the 12 classes. The green class is absent because green radish samples were not available during this additional collection.

### 2.3. Oriented-Object Sensing Output

The detection model is YOLO11s-OBB. For each visible object, the model outputs

(3) z=(c,x,y,w,h,θ,s),

where *c* is the class, (x,y) is the object center, *w* and *h* are the OBB side lengths, θ is the long-axis orientation and *s* is the detection confidence. The manually labeled OBB provides the reference value $z^{*}$. Detection performance is evaluated using OBB IoU and mAP, while orientation stability is evaluated using angle mean absolute error (MAE), standard deviation, maximum error and Acc@5°.

Image coordinates use the top-left corner of the cropped ROI as the origin, with the *x*-axis pointing right and the *y*-axis pointing down. OBB corner coordinates are stored as normalized image coordinates in the label files and converted back to pixels for angle evaluation. The angle is treated as a principal-axis direction modulo 180°; therefore, reversing the long edge does not change the physical direction used by the controller. In the YOLO11-OBB head, the class and confidence terms are derived from the class confidence branch. A separate objectness branch is not used in the MaixPy YOLO11-OBB output interface.

Because the orientation of an OBB has 180° periodic symmetry, the angle error between a predicted angle $\theta_{p}$ and a reference angle $\theta_{g}$ is defined as

(4) $$\Delta \theta =\left|\left(\left(\theta_{p}-\theta_{g}+90^{\circ }\right)\;\mathrm{mod}\;180^{\circ }\right)-90^{\circ }\right|.$$

This definition avoids discontinuity near $0^{\circ }$ and $180^{\circ }$ and is consistent with the principal-axis representation of elongated waste objects. For approximately square boxes, the principal-axis direction is less physically meaningful; therefore, angle errors are reported under several aspect-ratio thresholds.

**Figure 6:**
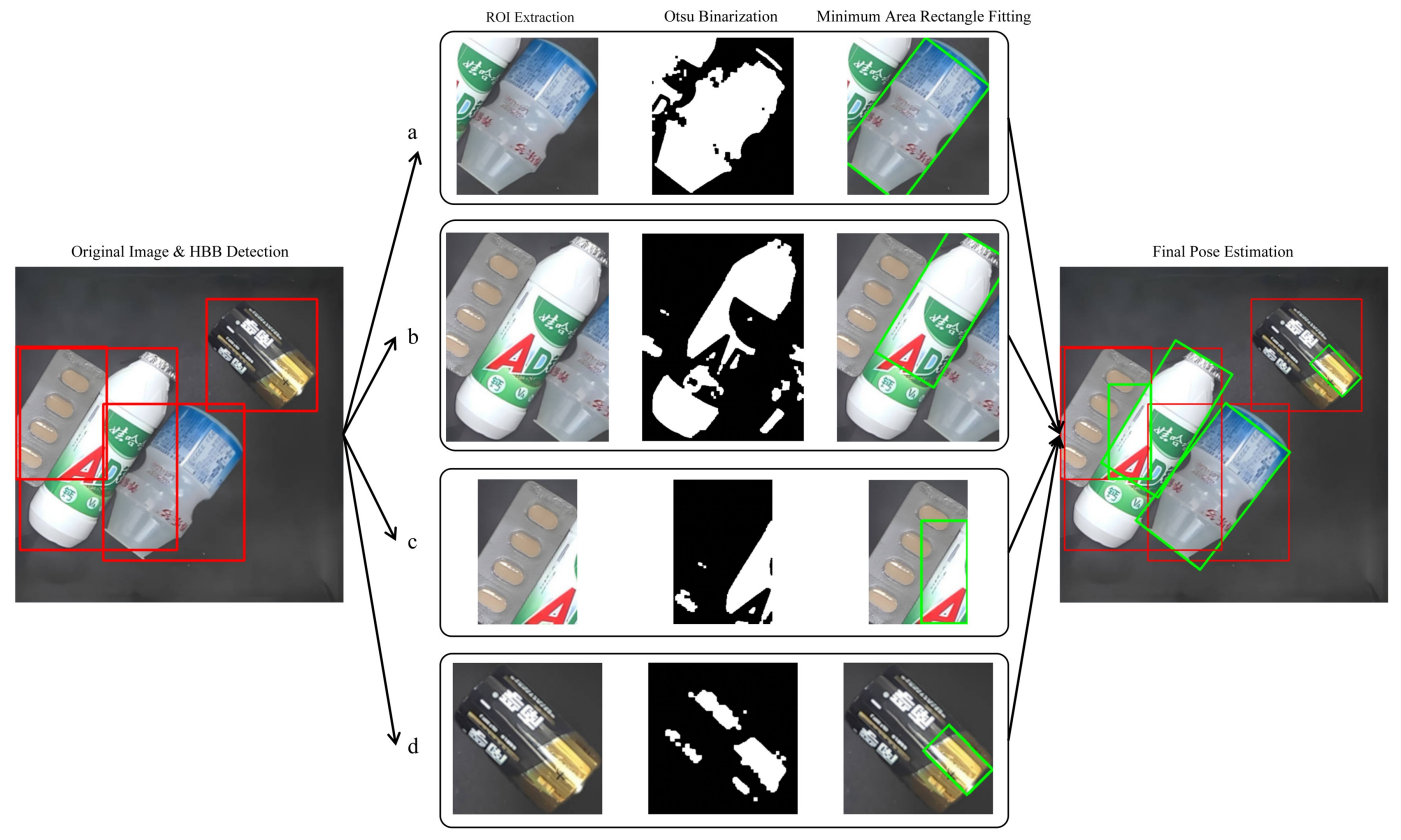
Illustrative outcomes of an HBB detection–segmentation–minimum-area-rectangle-fitting cascade: (**a**) a relatively isolated bottle with little contact with surrounding waste, for which most of the target contour is preserved and the fitted rectangle is comparatively accurate; (**b**) a bottle whose surface contains regions with insufficient color contrast, so only part of its contour is retained and the fitted rectangle does not cover the full object; (**c**) a blister pack of tablets in contact with an adjacent white bottle, where the target contour is largely lost after binarization and the rectangle is fitted mainly to the neighboring white region rather than to the target; and (**d**) a battery whose dark casing is similar to the background, so the fitted rectangle is dominated by the high-contrast terminal and covers only part of the object.

For waste sorting, an OBB output directly provides corners and principal-axis orientation. A cascade of HBB detection, region segmentation and minimum-area-rectangle fitting can also be used to estimate an angle, but its result depends on segmentation quality and contour extraction. The examples in Figure 6 compare a relatively isolated target with more difficult cases involving insufficient target contrast, loss of the target contour next to an adhering object, and similarity between a dark target and the background. These examples are qualitative illustrations and are not intended as a controlled segmentation baseline. OBB detection is used here because it gives the sensing module a direct rectangular orientation output and provides a compact interface for the sorting controller.

### 2.4. Class- and Aspect-Ratio-Aware Re-Sampling

The self-built dataset contains both class imbalance and elongated objects. Imbalanced and long-tailed training distributions can bias visual recognition models toward frequent categories [27]. Recent reviews on AI-based waste classification also identify dataset imbalance, limited environmental variation and inconsistent annotation as common barriers to deployment [3,4]. CAR-RS is used here as a simple image-level repeat-sampling heuristic. It is related to class-balanced and repeat-factor sampling, but it uses both object class counts and OBB aspect ratio because the downstream task depends on orientation as well as category. CAR-RS adjusts the sampling frequency of training images so that the model sees rare classes and high-aspect-ratio objects more often.

For the *i*th training image, let its target set be $G_{i}$. For an object *g*, the class is $c_{g}$ and the aspect ratio is

(5) $${\mathrm{AR}}_{g}=\frac{\max(w_{g},h_{g})}{\min(w_{g},h_{g})+\epsilon }.$$

In the implementation, $\epsilon =10^{-6}$ to avoid division by zero. The class count $n_{c_{g}}$ in Equation (6) denotes the number of labeled object instances of class $c_{g}$ in the original training set.

The class term is computed as

(6) $$W_{i}^{c}=\frac{1}{|G_{i}|}\sum_{g\in G_{i}}{\left(\frac{\bar{n}}{n_{c_{g}}}\right)}^{\beta },$$

where $n_{c_{g}}$ is the number of labeled object instances in class $c_{g}$, $\bar{n}$ is the average class count and β controls the class-balancing strength. The aspect-ratio term is defined as

(7) $$W_{i}^{\mathrm{AR}}=1+\gamma \cdot \min\left(2.0,\frac{1}{|G_{i}|}\sum_{g\in G_{i}}\max\left(0,{\mathrm{AR}}_{g}-\tau \right)\right),$$

where γ controls the aspect-ratio weight and τ is the aspect-ratio threshold. The final image-level sampling weight is

(8) $$W_{i}=W_{i}^{c}W_{i}^{\mathrm{AR}}.$$

The implementation uses τ=1.3, γ=0.8, β=0.5, a maximum repeat count of 3 for one image and a target list length of about 1.2 times the original training list. The weights are converted to repeat counts by normalizing all $W_{i}$ values by their mean, rounding to the nearest integer and clipping the result to the range [1,3]. If the expanded list is shorter than the target length, images with the largest fractional residuals are added until the target length is reached; if it is longer, surplus entries are removed from the lowest-weight repeated entries. The final list is shuffled with seed 0 before training. The selected values are heuristic settings used to keep the expanded list moderate: the list length increases by about 20%, and no image can appear more than three times. The final setting expanded the training list from 477 to 572 entries. The expanded list only repeats existing image paths and does not synthesize new images or labels. Therefore, CAR-RS does not introduce additional annotation noise or increase on-device inference cost, but it can still increase overfitting risk when the number of physical object instances is small. This risk is examined using the external cross-background test described in Section 3.4.

### 2.5. Class- and Aspect-Ratio-Aware Calibration

The INT8 model on MaixCAM Pro is generated by post-training quantization (PTQ). PTQ thresholds depend on the activation distributions produced by calibration images. Insufficient calibration coverage may reduce detection and angle-regression accuracy. This effect is consistent with journal studies on model compression and neural network quantization, which report that the activation distribution observed during calibration strongly affects integer inference behavior [31,32]. If the calibration set is dominated by common classes or nearly square objects, the branches related to rare or elongated objects may become less stable after quantization.

CAR-Calib selects calibration images only from the training image pool using class coverage, aspect-ratio coverage and scale coverage. No validation or external-test images are used for calibration. The aspect-ratio bins are [1.0,1.2), [1.2,1.5), [1.5,2.0) and [2.0,+∞). The scale bins are based on normalized OBB area: small for area <0.02, medium for 0.02≤ area <0.08 and large for area ≥0.08. For candidate image *i*, let $C_{i}$, $A_{i}$ and $S_{i}$ denote its class, aspect-ratio-bin and scale-bin sets. Let $N_{C}\left(c\right)$, $N_{A}\left(a\right)$ and $N_{S}\left(s\right)$ denote the current selected counts. The greedy score is

(9) $$Q_{i}=\sum_{c\in C_{i}}\frac{3.0}{\sqrt{N_{C}\left(c\right)+1}}+\sum_{a\in A_{i}}\frac{1.5}{\sqrt{N_{A}\left(a\right)+1}}+\sum_{s\in S_{i}}\frac{1.0}{\sqrt{N_{S}\left(s\right)+1}}+0.2M_{i},$$

where $M_{i}$ is the number of objects with AR≥1.5 in image *i*. In each round, the image with the highest score is selected and the coverage counts are updated until the calibration set reaches 100 images.

The coefficients in Equation (9) give the highest priority to class coverage, followed by aspect-ratio coverage and scale coverage. This ordering reflects the deployment goal: the INT8 calibration set should first cover all target categories, and then include elongated and differently sized objects that are more sensitive to angle estimation after quantization. The small $0.2M_{i}$ term is used only as a tie-breaking preference for images with multiple elongated objects.

**Figure 7:**
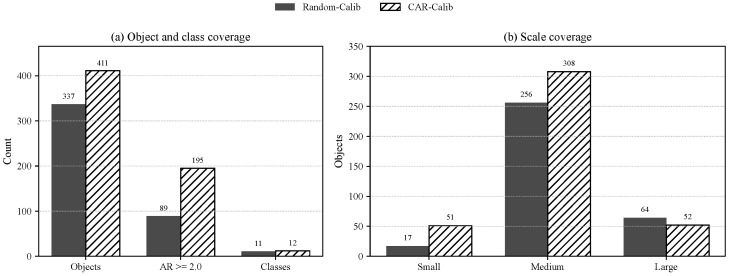
Coverage comparison between random calibration and CAR-Calib.

CAR-Calib does not modify model weights. It changes only the image subset used to estimate PTQ thresholds. The selected CAR-Calib set contains 100 images and 411 objects, including 195 objects with AR≥2.0. The random calibration set also contains 100 images but has 337 objects. This means the comparison changes both the coverage pattern and the number of object instances seen during calibration; the result is therefore treated as deployment evidence rather than a controlled causal study of the selection rule. The coverage comparison is shown in Figure 7.

### 2.6. ONNX Output-Node Adaptation for Embedded Sensing

On-device sensing uses the native MaixPy 4.10.3 nn.YOLO11 OBB interface. The trained PyTorch weights are first exported to an ONNX model that satisfies the MaixPy YOLO11-OBB parsing requirements [29,34]. The model is then converted to BF16 or INT8 cvimodel using TPU-MLIR 1.18.1 in a Docker Desktop 4.75.0 environment [30]. Finally, a mud description file is prepared and the model is loaded on MaixCAM Pro.

**Figure 8:**
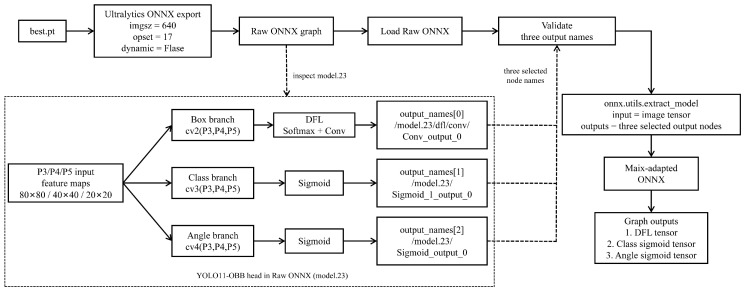
ONNX output-node adaptation before YOLO11-OBB deployment.

The ONNX adaptation process is shown in Figure 8. The model is exported with a fixed input size of 640×640, ONNX opset 17 and dynamic shapes disabled. The YOLO11-OBB head tensors are located in the ONNX graph. The selected outputs include the DFL branch, the class-confidence sigmoid branch and the angle sigmoid branch. A subgraph is extracted using the image input tensor and the selected head tensors as graph boundaries, producing a Maix-adapted ONNX model for TPU-MLIR compilation. BF16 conversion uses this ONNX model directly, while INT8 conversion uses calibration images to generate quantization thresholds.

The adapted ONNX graph has one input tensor, images, with shape [1,3,640,640]. The three exposed output tensors are:

- /model.23/dfl/conv/Conv_output_0: [1,1,4,8400];
- /model.23/Sigmoid_1_output_0: [1,12,8400];
- /model.23/Sigmoid_output_0: [1,1,8400].

The 8400 positions correspond to the flattened prediction locations from the three detection feature levels. The default exported output was not used because the MaixPy YOLO11-OBB parser expects these head tensors rather than a fused post-processed output. Therefore, graph extraction changes only the exposed ONNX outputs; the detector decoding, confidence thresholding and rotated-box post-processing are handled by the MaixPy runtime.

The mud file describes the model type, input format, normalization parameters and class labels required by the runtime. It sets the model type to yolo11, the detection type to obb and the input type to rgb, together with the 12 waste labels. During MaixCAM Pro execution, the program reads camera frames, calls detector.detect and exports or draws predicted class, confidence, OBB points and angle.

The conversion command uses TPU-MLIR model_transform.py with RGB/NCHW input, mean (0,0,0) and scale (1/255,1/255,1/255). BF16 deployment is generated by model_deploy.py –quantize BF16. INT8 deployment first runs run_calibration.py on 100 calibration images and then runs model_deploy.py –quantize INT8 –quant_input with the generated calibration table. The mud configuration and conversion scripts are provided in the Supplementary Minimal Dataset.

## 3. Results

### 3.1. Experimental Settings

PC-side training is implemented with Ultralytics YOLO11-OBB. The input size is 640×640, the number of epochs is 40 and the batch size is 16. The pretrained checkpoint is yolo11s-obb.pt. Training uses the Ultralytics automatic optimizer setting, which selected AdamW with an initial learning rate of 0.000625, momentum of 0.9 and weight decay of 0.0005. The warm-up period is three epochs. Mosaic augmentation is enabled during training and closed during the last ten epochs; horizontal flip probability is 0.5, mixup and copy-paste are disabled, and the random seed is 0. The loss weights are the default Ultralytics OBB settings: box 7.5, class 0.5 and DFL 1.5. Training and validation were performed on an NVIDIA GeForce RTX 4060 Laptop GPU (NVIDIA Corporation, Santa Clara, CA, USA) using Python 3.10.17, Ultralytics 8.3.215, PyTorch 2.7.0+cu118, CUDA 11.8, OpenCV 4.10.0, ONNX 1.17.0 and ONNX Runtime 1.22.0. The MMRotate experiments used PyTorch 2.0.1+cu118, MMCV 2.0.1, MMEngine 0.10.7, MMDetection 3.1.0 and MMRotate 1.0.0rc1.

The evaluation metrics include mAP50, mAP50–95, angle MAE, angle-error standard deviation, maximum angle error and Acc@5°. On-device testing is performed on MaixCAM Pro using 100 validation images. Predictions exported from actual on-device inference are matched with OBB labels. Precision, recall, F1-score and mAP50 are calculated following common object-detection evaluation practice [20,35]. The angle error is calculated using Equation (4). On-device matching uses greedy same-class OBB IoU matching with an IoU threshold of 0.5: all candidate prediction–ground-truth pairs are sorted by IoU, and the highest-IoU unused pair is selected first. Angle error is calculated only for matched true-positive detections, so it represents conditional orientation accuracy among detected objects. False negatives and false positives are reflected in precision, recall, F1-score and mAP.

To provide non-YOLO OBB baselines, the same training and validation labels were also converted to DOTA-style quadrilateral annotations [36]. Oriented R-CNN [37] and Rotated RetinaNet, implemented in MMRotate [38] with a ResNet-50 FPN backbone, were trained for 40 epochs at the same input size. Rotated RetinaNet follows the dense one-stage RetinaNet design with focal loss [39]. These baselines are used only for PC-side OBB comparison. Their MMRotate outputs require detector-specific decoding and rotated NMS, and they are not parsed by the native MaixPy nn.YOLO11 OBB interface used for the deployed model.

The choice of YOLO11s-OBB is tied to the target sensing task and deployment stack. The sorting controller requires class, location, object size and in-plane orientation from one model output. General object detectors such as RT-DETR, Faster R-CNN or RetinaNet can provide strong HBB detection baselines, but they do not directly provide the OBB angle required by the actuator. Rotated-detector families such as Oriented R-CNN or Rotated RetinaNet are useful OBB baselines in remote-sensing benchmarks, but they require a different training and conversion stack and are not supported by the native MaixPy nn.YOLO11 OBB parser used on MaixCAM Pro. Therefore, the embedded deployment comparison in this work is made within the deployable YOLO11-OBB path, while the MMRotate models are reported as PC-side OBB references.

### 3.2. PC-Side Training Results

Table 1 reports the PC-side validation results. Both models are trained for 40 epochs with the same input size and optimizer settings. CAR-RS only changes the sampling frequency in the training list. It does not change the network architecture or the on-device inference graph.

Both configurations obtain high mAP50. Since mAP integrates confidence ranking, classification and localization, it does not directly quantify the accuracy of the predicted orientation angle among matched detections. The angle error is therefore further evaluated by aspect-ratio groups, by the external cross-background test and by on-device quantized inference.

Table 2 reports two non-YOLO OBB baselines trained on the same image split after converting the labels to DOTA-style quadrilateral annotations. The MMRotate DOTA metric is an OBB AP metric under the DOTA/VOC evaluation protocol; it should not be read as COCO mAP50–95. Oriented R-CNN obtains 0.992 DOTA OBB mAP at epoch 19, while Rotated RetinaNet obtains 0.975 at epoch 26. The two MMRotate baselines therefore provide PC-side OBB references outside the YOLO family. However, these models are not used for MaixCAM Pro deployment because their output decoding and rotated NMS are not supported by the native MaixPy YOLO11-OBB parser.

**Figure 9:**
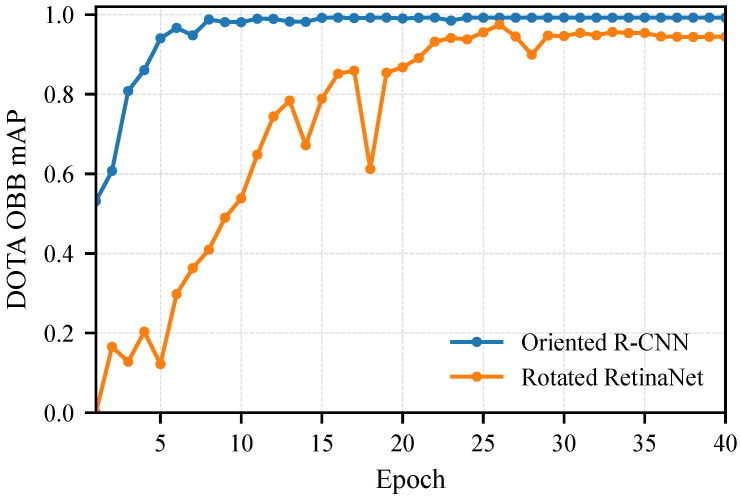
Validation DOTA OBB mAP curves of the two non-YOLO OBB baselines.

Figure 9 shows the validation mAP curves of the two MMRotate baselines. Oriented R-CNN converges faster on this small dataset, whereas Rotated RetinaNet reaches its best validation result later and shows larger fluctuations before convergence.

**Figure 10:**
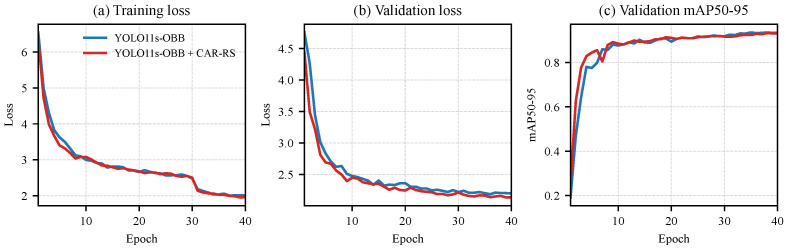
Training loss, validation loss and validation mAP50–95 curves on the PC-side validation set.

Figure 10 shows the training loss, validation loss and validation mAP50–95 curves for the baseline and CAR-RS settings. Both curves converge within 40 epochs. CAR-RS slightly reduces the validation loss during most of the training process, while the final global mAP50–95 is close to the baseline. This agrees with the table results: CAR-RS is not used to change the network capacity, but to improve the exposure of rare and elongated targets.

### 3.3. Aspect-Ratio Grouped Orientation Error

Table 3 lists the angle errors under different aspect-ratio thresholds. The object count is the number of validation objects satisfying the corresponding threshold. For the same threshold, both models are evaluated on the same object set. When AR≥1.3, YOLO11s-OBB + CAR-RS obtains an angle MAE of $3.201^{\circ }$, a standard deviation of $5.307^{\circ }$, a maximum error of $42.319^{\circ }$ and an Acc@5 of 86.17%.

For AR≥1.0 and AR≥1.3, CAR-RS improves MAE, standard deviation, maximum error and Acc@5. For AR≥1.5 and AR≥2.0, CAR-RS improves Acc@5 but gives a larger MAE than the baseline. These nested threshold groups are therefore interpreted cautiously rather than as independent groups. CAR-RS is treated as a mild data-level sampling strategy and is further evaluated under the external cross-background test and on-device quantized deployment.

**Figure 11:**
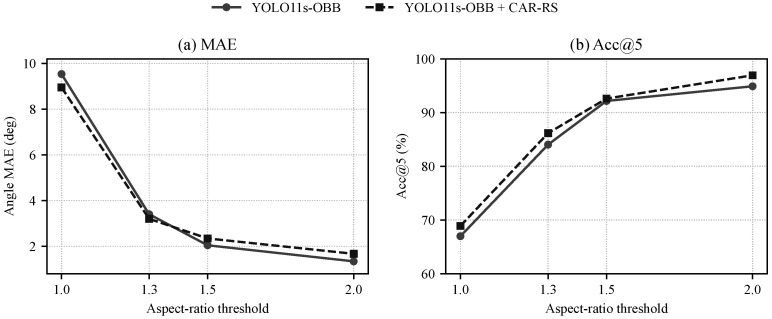
Angle MAE and Acc@5 under different aspect-ratio thresholds.

Figure 11 visualizes the MAE and Acc@5 trends. As the AR threshold increases, the number of evaluated objects decreases, MAE generally decreases and Acc@5 generally increases. This pattern is expected because near-square OBBs have ambiguous principal axes; the grouped results therefore separate angle reliability for elongated objects from the larger errors caused by less directional objects.

### 3.4. External Cross-Background Test

To address the limited scene variation in the original split, an additional cross-background test set was collected after model training. The set contains 80 images and 201 OBB annotations captured on the same MaixCAM Pro platform but with changed backgrounds and moderately different camera viewpoints. The set was not used for training, CAR-RS list construction, calibration or model selection. It contains 11 classes; the green class is absent because green radish samples were not available during this additional acquisition.

Table 4 reports the results of the original baseline and the CAR-RS model on this additional set. The accuracy is lower than on the original PC validation set, confirming that the original split was optimistic because of repeated object appearances and similar capture conditions. CAR-RS improves several cross-background measures: precision increases from 0.839 to 0.907, recall increases from 0.882 to 0.893, mAP50 increases from 0.916 to 0.927, and the number of matched elongated objects increases from 111 to 121. The stricter mAP50–95 metric changes from 0.797 to 0.789, and the angle MAE at AR≥1.3 decreases from $5.595^{\circ }$ to $5.234^{\circ }$.

**Figure 12:**
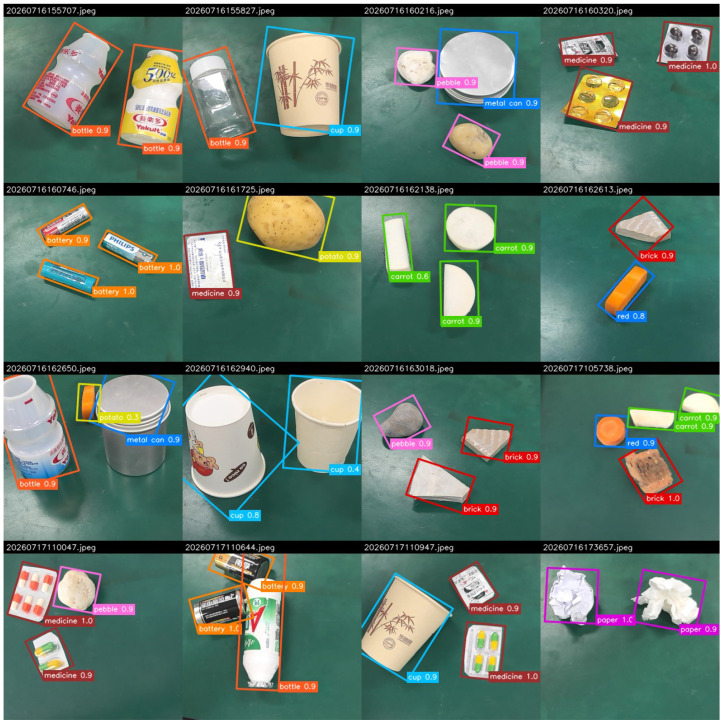
Representative OBB predictions on the additional cross-background test set.

Figure 12 shows representative predictions from the additional test set. The test is still limited in scale, but it gives a more conservative estimate than the original validation split and is therefore used in the discussion to qualify the generalization claim.

### 3.5. On-Device Quantization and Detection Results

Table 5 reports the 100-image MaixCAM Pro validation results. All results are calculated by matching prediction CSV files exported from actual on-device inference with local OBB labels. The results therefore include the effects of model conversion, quantization and MaixPy runtime execution.

YOLO11s-OBB + CAR-RS + CAR-Calib INT8 obtains the highest precision, F1-score and mAP50 in the on-device test, and ties for the highest recall. The difference between Random-Calib and CAR-Calib is small and is based on one selected calibration subset, so it is reported as a deployment observation rather than as a statistically established improvement. The BF16 model gives the lowest angle MAE, but it has a larger model size and lower FPS. For the current low-power platform, INT8 provides the more favorable speed and memory trade-off.

Table 6 lists the per-class AP50 of the YOLO11s-OBB + CAR-RS + CAR-Calib INT8 model. Some classes contain only a few objects in the 100-image on-device validation set, such as metal can and green. Their AP50 values should therefore be interpreted only as descriptive counts from this small controlled test, not as stable class-level estimates.

Table 7 gives the speed results from a fixed 30 s benchmark on MaixCAM Pro. The INT8 models have a size of about 11.8 MB and reach 8.40 FPS. The BF16 model has a size of 25.67 MB and runs at 1.99 FPS. Considering detection accuracy, angle error, model size and inference speed together, CAR-RS + CAR-Calib INT8 is selected as the deployment configuration. The benchmark counts camera-based model inference and display operation in the test program; it does not decompose preprocessing, neural-network execution, post-processing, serial transmission and actuator motion.

**Figure 13:**
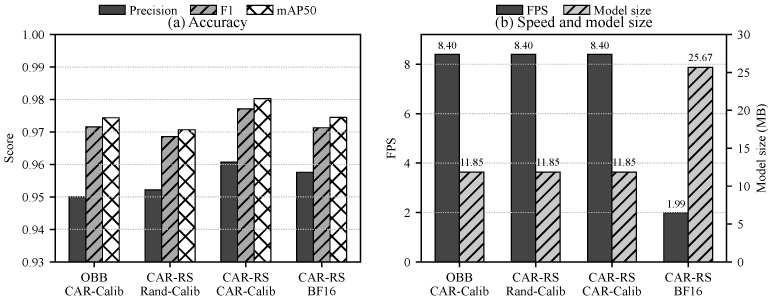
On-device detection accuracy, orientation error, speed and model size.

Figure 13 summarizes on-device mAP50, F1-score, angle MAE, FPS and model size. CAR-RS + CAR-Calib INT8 obtains high mAP50 and F1-score while maintaining 8.40 FPS. BF16 provides a lower angle MAE but has a larger model size and much lower FPS, showing the trade-off between numerical precision and embedded throughput on this device.

### 3.6. On-Device Sensing Visualization and Sorting Execution

**Figure 14:**
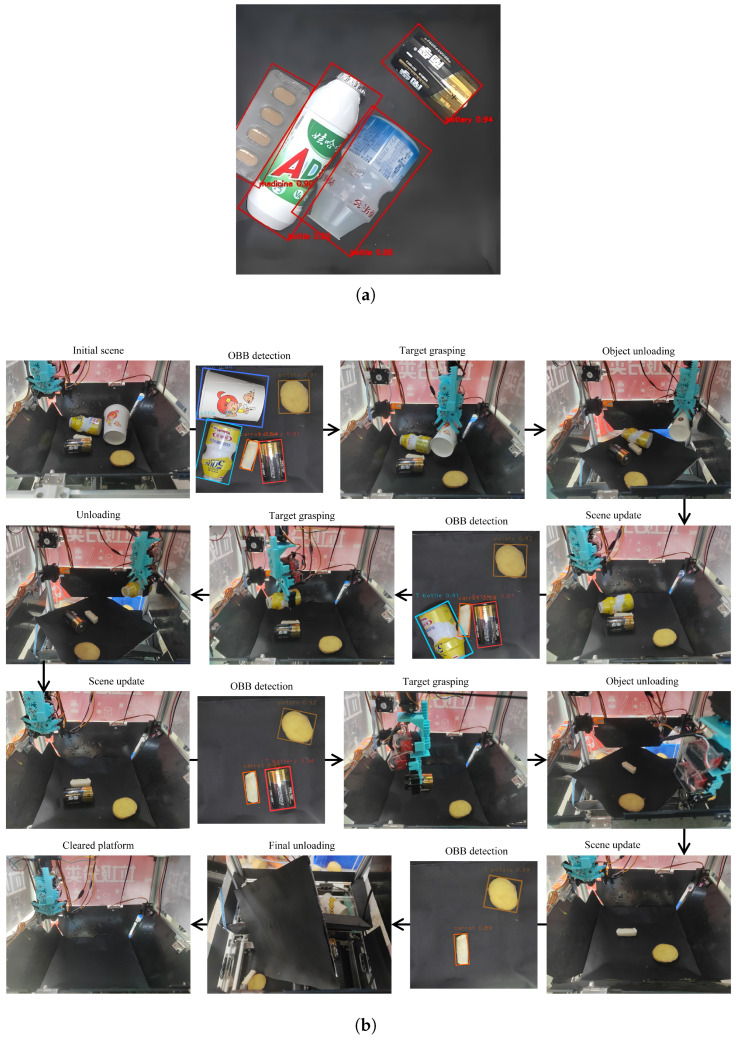
Visual output and sorting execution after MaixCAM Pro deployment. (**a**) On-device OBB detection output. (**b**) Sequential perception-action sorting process under multiple-object input.

Figure 14 shows on-device OBB sensing and a representative sorting execution sequence. Figure 14a shows the OBB output of MaixCAM Pro, including rotated boxes, class names and confidences. Figure 14b shows the perception-action process under multi-object input: the system detects the current platform state, selects a target, drives the actuator for grasping and unloading, and then reacquires the updated scene.

In addition to the visual sequence, a 50-round practical sorting record was summarized to check whether the deployed sensing output could support physical sorting actions on the prototype. The record contains 123 placed targets. Seven rounds were marked unsuccessful because one target in each round was assigned to an incorrect four-category sorting destination. Counting one target-level sorting-decision error for each failed round gives 116 correct target-level sorting decisions out of 123 targets, or 94.3%. At the round level, 43 of 50 sorting rounds were completed successfully, corresponding to a practical sorting success rate of 86.0%. Five rounds required a second grasping attempt. These values are reported as prototype operation records rather than as a large-scale per-class sorting benchmark.

## 4. Discussion

The deployed detector is used as a sensing module rather than as a standalone vision benchmark. Its output is a class label, an image-plane center, an OBB extent and a principal-axis angle. These values are the quantities sent to the Mega2560 controller after the four-frame stability check. The current pixel-to-controller mapping is sufficient for the small prototype, but it is not a calibrated camera-to-actuator transformation. Therefore, the reported 8.40 FPS and detection metrics should be read as visual-sensing and embedded inference results, not as proof of a fully characterized robotic sorting cycle.

Most waste-detection studies still report category classification or HBB detection accuracy. Recent work has improved lightweight waste detection, plastic or recyclable waste recognition and difficult-scene object detection [8,9,10,11,12,13,14,17]. Those results are relevant to the recognition part of the problem. The present work adds a different constraint: the detector output must retain an in-plane orientation and remain compatible with the MaixCAM Pro conversion path. This is why the paper evaluates OBB angle error, output-node adaptation, PTQ deployment and on-device runtime in addition to mAP.

The non-YOLO OBB baselines further separate two questions that are often mixed together: whether the dataset can be learned by other oriented detectors, and whether the resulting model can be deployed on the target camera. Oriented R-CNN and Rotated RetinaNet both obtain high PC-side DOTA OBB AP on the converted validation set, so the choice of YOLO11-OBB is not based only on validation accuracy. The reason for using YOLO11-OBB in the deployed system is the available MaixPy OBB runtime: it can parse the adapted YOLO11 OBB head and return class labels, OBB points and angles directly. The MMRotate baselines would require a separate embedded decoding path and rotated NMS implementation before they could be evaluated fairly on MaixCAM Pro.

CAR-RS and CAR-Calib were kept outside the network architecture. This matters for MaixCAM Pro because additional custom layers or CPU post-processing would increase the risk of conversion failure. CAR-RS does not improve every metric: on the original validation split it slightly reduces mAP50–95 and worsens angle MAE for the stricter AR≥1.5 and AR≥2.0 cumulative subsets. Its clearer benefit appears on the added cross-background set, where mAP50 increases from 0.916 to 0.927 and the number of matched elongated objects increases from 111 to 121, although mAP50–95 changes from 0.797 to 0.789. The angle MAE for matched objects with AR≥1.3 decreases from $5.595^{\circ }$ to $5.234^{\circ }$. This external result is still small, but it is a better test of whether the repeat-sampling list helps beyond the original near-duplicate validation split.

The BF16 and INT8 results show a practical deployment trade-off on the tested device. BF16 gives the lowest angle MAE in Table 5, but the measured throughput is only 1.99 FPS. INT8 reduces the model file to about 11.85 MB and reaches 8.40 FPS. The CAR-Calib INT8 result is slightly better than the tested random-calibration INT8 result in mAP50 and F1-score. This should not be over-interpreted, because only one random calibration subset was tested and the CAR-Calib subset contains more object instances. The result is best viewed as evidence that calibration-set composition should be reported and controlled when an OBB detector is quantized.

The ONNX adaptation is also runtime-specific. The adapted model exposes the DFL, class-sigmoid and angle-sigmoid tensors expected by the MaixPy nn.YOLO11 OBB parser. This avoids a custom OBB decoder in the Python application, but it also ties the conversion script to the tensor conventions of the current Ultralytics and MaixPy implementations. A different detector family or a future parser version would require the output names, tensor shapes and post-processing assumptions to be checked again.

The main threat to validity is the dataset itself. The original split contains repeated physical objects and similar backgrounds, so near-perfect PC-side mAP values are expected and should not be generalized to open-world waste streams. The added 80-image cross-background set gives a more conservative estimate of external-scene performance and makes this issue visible. Further evaluation should use new object instances, uncontrolled lighting, partial occlusion, contamination, specular surfaces and motion blur. The next hardware step is to measure the relation between image-plane error and mechanical tolerance: pixel localization error, physical positioning error, grasp success, wrong-bin drops, cycle time and recovery behavior should be recorded in the same trial log.

## 5. Limitations

The original dataset used in this study was collected on a self-built platform under similar camera and lighting conditions. Although the training and validation sets are separated by file list, some target objects have similar appearances across the two splits. The PC-side mAP values on that split should therefore be interpreted as validation results under the current acquisition setting, not as evidence of broad scene generalization. The additional 80-image cross-background test set reduces this risk, but it is still small and does not include the green class. A larger cross-scene test set with new object instances, stronger lighting variation and a full 12-class coverage is still needed. The number of unique physical items per class and acquisition-session identifiers were not recorded in the original dataset metadata, so the paper reports image counts and object annotation counts rather than claiming object-instance-disjoint evaluation.

The non-YOLO OBB baselines in this paper are PC-side comparisons only. They were trained and evaluated after converting the labels to DOTA-style annotations, but they were not converted to MaixCAM Pro models. A direct on-device comparison would require custom parsers for their output tensors, rotated box decoding and rotated NMS. This is beyond the current native MaixPy YOLO11-OBB deployment path and remains future work.

The present system covers 12 classes used by the experimental sorting platform. The carrot, green and red labels denote white radish, green radish and carrot/yellow radish samples, respectively, rather than universal waste categories. Other waste objects can be added by collecting new OBB labels and updating the class list, but the current results should not be read as a complete municipal-waste taxonomy. The mechanical sorting strategy is also simple: it uses detected class, area and pose to drive grasping or unloading. The 50-round operation record provides an initial check of physical execution on the prototype, but it is still not a controlled per-class success-rate benchmark. Future work should record larger per-class sorting trials, including missed grasps, wrong-bin drops and recovery actions.

The camera-to-actuator geometry is not fully calibrated in the present prototype. The paper reports the ROI, coordinate convention and pixel-to-controller mapping used in the code, but camera intrinsic parameters, lens distortion, metric pixel-to-millimetre conversion and error propagation were not measured. For a production sorting machine, these terms should be calibrated and connected to mechanical tolerance and actuator success rate.

The architecture comparison is limited by the embedded OBB deployment toolchain. Non-YOLO detectors and rotated two-stage detectors are relevant research baselines, but they cannot be executed through the current native MaixPy YOLO11-OBB runtime without a separate conversion and post-processing pipeline. For this reason, the study focuses on improving and evaluating the deployable YOLO11-OBB path. Future work will compare additional OBB detector families when a common embedded conversion path is available.

## 6. Conclusions

This study demonstrates that a YOLO11s-OBB detector can be converted to a MaixPy-compatible graph and executed on MaixCAM Pro as an embedded waste-sorting perception module. On the original validation split, the baseline and CAR-RS models both reached 0.995 mAP50; the baseline had the higher mAP50–95, whereas CAR-RS gave a smaller conditional angle MAE at AR≥1.3 ($3.403^{\circ }$ to $3.201^{\circ }$). The added 80-image cross-background test was more demanding than the original validation split, and CAR-RS improved precision from 0.839 to 0.907, recall from 0.882 to 0.893 and mAP50 from 0.916 to 0.927, although mAP50–95 changed from 0.797 to 0.789. On MaixCAM Pro, the selected INT8 model reached 0.961 precision, 0.994 recall, 0.977 F1-score and 0.980 mAP50 on the 100-image on-device validation set, with an 11.85 MB model file and 8.40 model inferences per second under the reported benchmark conditions.

The evidence supports the feasibility of OBB-based embedded visual sensing on the prototype. The 50-round practical sorting record further indicates that the sensing output can support physical sorting actions in the tested setup, but it does not yet establish broad sorting robustness. Generalization to unseen object instances and scenes, calibrated camera-to-actuator accuracy, end-to-end latency and larger per-class sorting trials remain future work.

## Figures and Tables

**Table 1:** PC-side validation results.

Model Configuration	Training Entries	Best Epoch	mAP50	mAP50–95
YOLO11s-OBB	477	35	0.995	0.935
YOLO11s-OBB + CAR-RS	572	38	0.995	0.932

**Table 2:** PC-side non-YOLO OBB baseline results using MMRotate.

Model	Framework and Evaluator	Best Epoch	DOTA OBB mAP	DOTA AP50
Oriented R-CNN R50-FPN	MMRotate DOTA	19	0.992	0.992
Rotated RetinaNet R50-FPN	MMRotate DOTA	26	0.975	0.974

**Table 3:** Angle errors under different aspect-ratio thresholds.

Method	AR Threshold	Objects	MAE (°)	Std (°)	Max (°)	Acc@5 (%)
YOLO11s-OBB	1.0	418	9.537	17.246	89.513	66.99
+ CAR-RS	1.0	418	**8.949**	**15.977**	**88.638**	**68.90**
YOLO11s-OBB	1.3	282	3.403	6.328	51.868	84.04
+ CAR-RS	1.3	282	**3.201**	**5.307**	**42.319**	**86.17**
YOLO11s-OBB	1.5	230	**2.045**	**2.807**	**23.784**	92.17
+ CAR-RS	1.5	230	2.344	4.026	42.319	**92.61**
YOLO11s-OBB	2.0	98	**1.343**	**1.523**	**9.225**	94.90
+ CAR-RS	2.0	98	1.672	2.600	22.930	**96.94**

**Table 4:** External cross-background test results on the 80-image set.

Model	Precision	Recall	mAP50	mAP50–95	Matched Objects (AR≥1.3)	Angle MAE (AR≥1.3)
YOLO11s-OBB	0.839	0.882	0.916	**0.797**	111/136	5.595°
YOLO11s-OBB + CAR-RS	**0.907**	**0.893**	**0.927**	0.789	**121/136**	**5.234°**

**Table 5:** MaixCAM Pro 100-image on-device validation results.

Method	Precision	Recall	F1-Score	mAP50	Angle MAE (°)
YOLO11s-OBB + CAR-Calib INT8	0.950	0.994	0.972	0.974	10.165
YOLO11s-OBB + CAR-RS + Random-Calib INT8	0.952	0.986	0.969	0.971	9.879
YOLO11s-OBB + CAR-RS + CAR-Calib INT8	**0.961**	**0.994**	**0.977**	**0.980**	9.786
YOLO11s-OBB + CAR-RS + BF16	0.958	0.986	0.971	0.975	**9.357**

**Table 6:** Per-class AP50 of the YOLO11s-OBB + CAR-RS + CAR-Calib INT8 model.

Class	GT Objects	AP50	Class	GT Objects	AP50
bottle	29	0.964	pebble	42	1.000
cup	12	1.000	brick	32	1.000
battery	96	1.000	potato	12	1.000
metal can	4	1.000	carrot	40	1.000
medicine	36	1.000	green	5	0.800
red	24	1.000	paper	12	1.000

**Table 7:** Model size and inference speed on MaixCAM Pro.

Deployment Format	Model Size (MB)	Average FPS	Note
BF16	25.67	1.99	Higher numerical precision
INT8 + Random-Calib	11.84	8.40	Random calibration set
INT8 + CAR-Calib	11.85	**8.40**	Selected deployment setting

## Data Availability

The data supporting the main findings of this study are contained within the article and the Supplementary Material. The Supplementary Minimal Dataset includes processed experimental records, training and calibration lists, conversion scripts, the additional cross-background test labels, the physical sorting operation record and the on-device evaluation files used to generate the reported tables and figures. Additional raw capture files can be made available by the corresponding authors upon reasonable request.

## Supplementary Materials

The following supporting information can be downloaded at: https://www.mdpi.com/article/10.3390/s26154786/s1, Supplementary File S1: Minimal reproducibility dataset (ZIP archive) containing the OBB training and validation data and labels, the 80-image cross-background test set, training and calibration lists, processed experimental records, model-conversion and evaluation scripts, physical sorting operation records, and on-device evaluation files.

## Abbreviations

The following abbreviations are used in this manuscript:

AP	Average precision
AR	Aspect ratio
BF16	bfloat16 brain floating-point format
CAR-Calib	Class- and aspect-ratio-aware calibration
CAR-RS	Class- and aspect-ratio-aware re-sampling
DFL	Distribution focal loss
FPS	Frames per second
HBB	Horizontal bounding box
INT8	8-bit integer
IoU	Intersection over union
MAE	Mean absolute error
OBB	Oriented bounding box
ONNX	Open Neural Network Exchange
PTQ	Post-training quantization
